# Early bare-metal stent thrombosis presenting with cardiogenic shock: a case report

**DOI:** 10.1186/1752-1947-5-509

**Published:** 2011-10-08

**Authors:** Konstantinos M Lampropoulos, Themistoklis A Iliopoulos, Werner Budts

**Affiliations:** 1 Cardiology Department, Catheterization Laboratory 251 General Air Force Hospital, Athens, Greece; 2 Department of Cardiology, University Hospitals Leuven, Leuven, Belgium

## Abstract

**Introduction:**

Although stents have improved the safety and efficacy of percutaneous coronary interventions, coronary stent thrombosis remains a serious complication.

**Case presentation:**

We present the case of a 64-year-old Caucasian man from Greece, with symptoms and electrocardiographic findings suggestive of acute inferior myocardial infarction, who complained of chest pain and rapidly developed cardiogenic shock 48 hours after primary percutaneous coronary intervention.

**Conclusion:**

The most common cause of early bare-metal stent thrombosis is stent malapposition. Intravascular ultrasound is the preferred method to recognize predictors of coronary events that are not detected by angiography.

## Introduction

Stents have improved the safety and efficacy of percutaneous coronary interventions (PCI) by reducing acute or imminent vessel closure and by reducing restenosis rates compared with conventional balloon angioplasty [1]. In addition, coronary vasomotor reactivity has been found intact after stent implantation and long-term clinical and angiographic follow-up have attested to the durability of their action [2]. Nevertheless, coronary stent thrombosis remains a serious complication of PCI.

## Case presentation

A 64-year-old male Caucasian patient was admitted to our hospital with clinical and electrocardiographical findings suggesting acute inferior myocardial infarction. Our patient had a history of hypertension and dyslipidemia but was not taking any medication at the time of admission. Laboratory findings were suggestive of acute cardiac ischemia. His plasma levels of N-terminal pro-B-type natriuretic, troponin I, creatine kinase and creatine kinase MB isoenzyme were increased. The first transthoracic echocardiogram executed at our emergency department showed hypokinesia of the inferior and posterior left ventricular wall. Our patient received 600 mg clopidogrel, 325 mg aspirin and 5000 U of unfractionated heparin and was then transferred to the catheterization laboratory, while receiving glycoprotein IIb/IIIa inhibitors (abciximab) intravenously.

**Figure 1:**
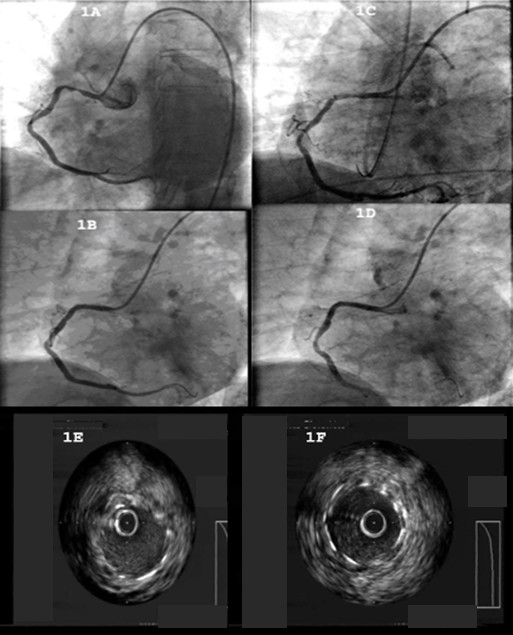
**Coronary angiography**. **(A) **The angiography showed: a severe stenosis (80-90%) at the proximal segment of the RCA and a second, moderate stenosis (40-50%) at the mid segment of the aforementioned vessel; **(B) **the RCA after primary PCI (TIMI III flow); **(C) **the RCA after thrombus inspiration with the PRONTO V3 device; **(D) **results after a balloon dilatation of the stent, which restored a TIMI III flow inside the vessel: **(E) **the study of the lesion using the IVUS, which showed the malapposition of the stent; **(F) **positive results after a balloon dilatation of the stent with IVUS study.

Coronary angiography showed atheromatosis of his left anterior descending artery and his left circumflex artery without any evidence of severe stenoses. There was one severe stenosis (80-90%) at the proximal segment of his right coronary artery (RCA) and a second, moderate stenosis (40-50%) at its mid segment (Figure 1A).

In view of the angiographic findings, primary PCI was performed. The intervention started with a predilatation of the severe lesion with a compliant balloon 2 × 20 mm at 10 Atm, followed by the deployment of a bare-metal chromium-cobalt stent 4 × 16 mm at 14 Atm. The final result was deemed satisfactory with TIMI III flow, and the fully expanded stent appropriately sized in length and diameter (Figure 1B). Our patient was subsequently transferred to the intensive care unit, where he remained hemodynamically stable for 24 hours while receiving, among other medications according to the American College of Cardiology/American Heart Association/European Society of Cardiology guidelines, glycoprotein IIb/IIa inhibitors (abciximab) intravenously.

Forty-eight hours after admission, our patient complained of chest pain and developed complete heart block and then asystole, suggestive of acute inferior myocardial infarction. Our patient went into cardiogenic shock. Inotropes and intravascular volume expander were given intravenously. A temporary pacemaker was placed through a central venous line. An intra-aortic balloon pump was also used. A new angiography showed total occlusion of the proximal segment of his RCA, with TIMI 0 flow. The intervention proceeded with aspiration of the thrombus using a PRONTO device (Figure 1C) and intravascular ultrasound (IVUS) of the culprit lesion showing malpositioning of the stent (Figure 1E). Finally, balloon dilatation of the stent and a postdilatation IVUS study took place (Figure 1F). The procedure successfully restored TIMI III flow in his RCA (Figure 1D). Our patient went on to a full and uneventful recovery after that and was discharge seven days later without any further complications.

## Discussion

Stent thrombosis is defined as an acute thrombotic occlusion in the stented segment of a coronary artery, usually presenting as ST-segment elevation myocardial infarction [1], and typically occurs within the first several weeks after stent placement. Stent thrombosis has traditionally been categorized as either subacute or early thrombosis, occurring within 30 days, or as late stent thrombosis, occurring later than 30 days [3]. While very late stent thrombosis, occurring beyond one year, is been increasingly described with the use of drug-eluting stents [3], such a thrombosis is rare with bare-metal stents.

Although early aggressive antiplatelet regimens were associated with unacceptably high rates of stent thrombosis and bleeding complications, the advent of dual antiplatelet therapy had salutary effects on both adverse outcomes.

However, in spite of the recent advancements in antiplatelet therapies, stent thrombosis is still recognized in 0.5-2% of elective cases, and in up to 6% of patients with acute coronary syndromes undergoing PCI [4].

Furthermore, longer stent lengths, large numbers of implanted stents, stent malapposition, residual dissections, reduced TIMI flow, gene polymorphisms and resistance to the antiplatelet effects of acetylsalicylic acid and thienopyridines are reported to increase the risk for stent thrombosis [1].

Previous studies have reported clinical and angiographic factors predictive of subacute stent thrombosis, including unstable angina, diabetes, age and long complex lesions [4]. However, these factors alone do not predict the possibility of periprocedural vessel closure in individual patients. IVUS provides unique, detailed qualitative and quantitative tomographic and transmural imaging of coronary lesions, both pre- and post-intervention.

The factors associated with a higher incidence of subacute stent thrombosis include patient age (> 65 years), tobacco use and ejection fraction (< 40%). On the other hand, factors associated with better outcome following stent thrombosis, are postprocedural TIMI III flow, residual stenosis < 50% and the use of glycoprotein IIb/IIIa inhibitors during and after PCI. The use of glycoprotein IIb/IIIa inhibitors is associated with a lower incidence of the "no reflow" phenomenon. Moreover, IVUS has the potential to recognize predictors of coronary events not detected by angiography.

## Conclusion

The most common cause of early bare-metal stent thrombosis is stent malapposition. This can be attributed to dissection at the edges of the stent or stent deployment issues. The latter include incomplete expansion (occurs when a portion of the stent is inadequately expanded, compared with the distal and proximal reference dimensions) and apposition (occurs when part of the stent is not fully in contact with the vessel wall, potentially increasing local flow disturbances) [1]. All of the above mentioned issues can be easily identified by IVUS which is the preferred method when assessing the anatomy of a lesion for sizing, position of plaque and adequacy of stent deployment [5].

## Consent

Written informed consent was obtained from the patient for publication of this case report and any accompanying images. A copy of the written consent is available for review by the Editor-in-Chief of this journal.

## Competing interests

The authors declare that they have no competing interests.

## Authors' contributions

KML and TAI contributed to the manuscript, performed the primary PCI and the IVUS study. KML and WB contributed to the manuscript, to the interpretation of the data and manuscript preparation. All authors read and approved the final manuscript.

## References

[B1] Grossman W Baim DS Grossman's Cardiac Catheterization, Angiography, and Intervention 2005 7 Lippincott Williams & Wilkins21987966

[B2] Ong AT McFadden EP Regar E de Jaegere PP van Domburg RT Serruys PW Late angiographic stent thrombosis (LAST) events with drug-eluting stents J Am Coll Cardiol 2005 45 12 2088 2092 10.1016/j.jacc.2005.02.086 15963413

[B3] Karvouni E Korovesis S Katritsis DG Very late thrombosis after implantation of Sirolimus eluting stent Heart 2005 91 6 e45 10.1136/hrt.2004.056341 15894746 PMC1768915

[B4] Mueller C Roskamm H Neumann FJ Hunziker P Marsch S Perruchoud A Buettner HJ A randomized comparison of clopidogrel and aspirin versus ticlopidine and aspirin after the placement of coronary artery stents J Am Coll Cardiol 2003 41 6 969 973 10.1016/S0735-1097(02)02974-1 12651043

[B5] Tobis J Azarbal B Salvin L Assessment of intermediate severity coronary lesions in the catheterization laboratory J Am Coll Cardiol 2007 49 8 839 848 10.1016/j.jacc.2006.10.055 17320741

